# Parental knowledge, attitudes and beliefs regarding fever in children: an interview study

**DOI:** 10.1186/s12889-016-3224-5

**Published:** 2016-07-11

**Authors:** Maria Kelly, Laura J. Sahm, Frances Shiely, Ronan O’Sullivan, Aoife McGillicuddy, Suzanne McCarthy

**Affiliations:** Pharmaceutical Care Research Group, School of Pharmacy, University College Cork, Cork, Ireland; Health Research Board Clinical Research Facility, Mercy University Hospital, Cork, Ireland; Department of Pharmacy, Mercy University Hospital, Cork, Ireland; Department of Epidemiology and Public Health, University College Cork, Cork, Ireland; School of Medicine, University College Cork, Cork, Ireland; National Children’s Research Centre, Dublin 12, Ireland; Department of Pharmacy, Cork University Hospital, Cork, Ireland

**Keywords:** Attitudes, Children, Fever, Knowledge, Parents

## Abstract

**Background:**

Fever is one of the most common childhood symptoms. It causes significant worry and concern for parents. Every year there are numerous cases of over- and under-dosing with antipyretics. Caregivers seek reassurance from a variety of sources including healthcare practitioners. The aim of this study was to describe parental knowledge, attitudes and beliefs regarding management of childhood fever in children aged 5 years and under.

**Method:**

Semi-structured interviews were conducted with 23 parents at six ante-natal clinics in the south west of Ireland during March and April 2015. The Francis method was used to detect data saturation and thereby identify sample size. Thematic analysis was used to analyse the data.

**Results:**

Twenty-three parents participated in the study. Five themes emerged from the data: assessing and managing the fever; parental knowledge and beliefs regarding fever; knowledge source; pharmaceutical products; initiatives. Parents illustrated a good knowledge of fever as a symptom. However, management practices varied between participants. Parents revealed a reluctance to use medication in the form of suppositories. There was a desire for more accessible, consistent information to be made available for use by parents when their child had a fever or febrile illness.

**Conclusion:**

Parents indicated that further initiatives are required to provide trustworthy information on the management of fever and febrile illness in children. Healthcare professionals should play a significant role in educating parents in how to manage fever and febrile illnesses in their children. The accessible nature and location of pharmacies could provide useful support for both parents and General Practitioners.

## Background

Fever is defined as “an elevation of body temperature above the normal daily variation” [1]. Normal temperature is described as between 36 and 36.8 °C [2]. Fever is one of the most common childhood symptoms [3–7] with up to 40 % of children under 6 months experiencing a fever [8]. Fever causes concern and anxiety in parents [6, 8–10] and healthcare professionals [6] alike, and is one of the main reasons parents seek reassurance and advice from healthcare professionals [11, 12]. Fever and febrile illness accounts for 6 % of visits to paediatricians, along with numerous visits to General Practitioners (GPs), emergency departments (EDs), primary care Paediatricians and out-of-hours care services [3, 5, 12–16].

Parental concern about childhood fever and consequent antipyretic use is increasing [17]. In the United States of America, consultations due to fever in children costs an estimated $10 billion annually, covering 60 million clinic visits [18–23]. Furthermore, analgesics and antipyretics account for a significant number of medication errors in children [24, 25]. Therefore, assessing parents’ knowledge and beliefs in managing fever and febrile illness is necessary so that safe and effective ways of managing fever can be communicated. This will potentially decrease unnecessary presentations at clinics and hospitals [26, 27] along with unintentional over- and under-dosing with antipyretics [6, 28].

The UK-based National Institute for Health and Care Excellence (NICE) Guideline Development Group on the assessment and management of feverish illness in children younger than 5 years recommended that a study be undertaken to investigate home-based antipyretic use and parental perception of distress in children with febrile illness (1). It was suggested that the study should include parents’ and carers’ interpretation of distress, including: help seeking behaviour, what triggers presentation to a healthcare professional, what triggers the decision to give a dose of antipyretic, and what triggers the decision to change from one antipyretic to another (1). Furthermore, a review of scientific literature indicated that parental knowledge regarding definition and management of fever is deficient [18, 29–32]. Studies from the United States of America, France, Palestine and Saudi Arabia have shown that parents rarely define fever correctly [18], are unaware of the correct frequency to administer antipyretics [29], have misconceptions regarding fever [30] and engage in practices which differ from recommendations [3, 5]. However, why parents have these misconceptions is unclear as in-depth interview studies to provide explanations for such practices are limited [10, 33, 34]. The purpose of this study was to examine the knowledge, attitudes and beliefs of parents regarding fever and febrile illness in children under 5 years of age.

## Method

### Design

A phenomenological approach was used to explore the lived experiences of parents when caring for a febrile child. Phenomenology examines human experiences through detailed descriptions of these experiences [35, 36]. It involves studying a small number of participants through in-depth engagement to develop patterns and assign meaning [35, 36]. The researcher excludes their own pre-conceived ideas and notions so that the experiences of the participants are elucidated from the research [35, 36]. Frameworks or other structures are not used in this type of research [35, 36]. Using this approach allowed for a deeper understanding of the lived experiences of parents [37, 38].

### Data collection

A topic guide for semi-structured interviews was developed based on the study objectives, drawing on existing literature and professional opinions. Table 1 summarises the final topics discussed.

**Table 1:** Topics discussed in interviews

Demographic information	
Identifying fever	
How to assess fever	
Normal and febrile temperatures	
Beliefs and thoughts about fever	
Practices used to manage fever	
Services used when child/children have fever?	
Knowledge about fever in general	
Where parents obtained their knowledge on fever	
Need for/form of additional resources	

Eligible participants were parents of children where at least one child was aged 5 years or younger at the time of the study. All interviews were conducted by MK, a research pharmacist not involved in the care of the children. Participants had no prior knowledge or relationship with the interviewer. The guide was piloted with eight parents who suggested modifications (decreasing number of questions asked and clarification of questions) based on their experiences with fever in children. Interviews took place in March and April 2015 in ante-natal clinics located in: Bantry General Hospital, Cork University Maternity Hospital, Mallow Primary Healthcare Centre, Mitchelstown Living Health Centre, St. Finbarr’s Hospital and St. Mary’s Health Campus. These clinics were selected to minimise selection bias, as they provide services to parents in the South West of Ireland, covering a wide geographical area and thus a wide range of demographics.

From the selected clinics, convenience sampling was undertaken; the primary researcher (MK) or a midwife invited participants who presented at the clinic on a given day to participate. Participants were free to decline participation in the study. No incentive to participate was offered. Written informed consent was obtained from participants.

The Francis method was used to guide the process of determining data saturation (when no new data occurred from a further three interviews) and hence sample size [39]. The point at which data saturation occurred determined sample size. Repeat interviews were not performed. Participants were not asked to review their transcripts. Field notes were made following each interview.

### Analysis

Thematic analysis was used to analyse the data [40]. Open coding of meaning units captured the knowledge, attitudes and beliefs of the participants. Inductive reasoning (coding) was used to construct salient categories of meaning and to identify relationships between categories derived from the data. Thematic analysis offered the opportunity to explain the social processes under study.

Data (transcripts and field notes) were entered into QSR International’s NVivo 10 Qualitative Data Analysis Software [41]. An inductive semantic approach to analysis was used to analyse the data [40].

The data were broken down into discrete incidents [42] and coded into categories. Two forms of categories were observed in the data: categories derived by the participant; and categories identified by the researcher as significant to the project’s focus of inquiry. Categories were exposed to content and definition changes as units were compared and as relationships between categories were developed or refined during analysis.

### Inter-coder reliability

A sample of three random transcripts were assessed by a co-author (AMG) in order to ensure coding consistency and aligned thinking between coders regarding the final themes. An inter-rater reliability score was calculated using Cohen’s Kappa by comparing coded transcripts from MK and AMG. Cohen’s Kappa scientifically measures the degree of agreement between coders and ranges from 0 to 1; 0 indicating no agreement and 1 indicating perfect agreement [43].

### Reporting

This study is reported following the Consolidated criteria for reporting qualitative studies (COREQ) guidelines [44].

## Results

The results presented here reflect the entire data set and provide a rich thematic description of the data so as to convey a sense of the predominant themes.

### Characteristics of respondents

Of the forty-six parents invited to participate in the interviews, 21 did not meet the inclusion criteria. Two parents declined to participate (due to lack of time) leaving 23 parents who participated in 21 interviews. If both parents were present and were willing to participate, then both were included in the interview (which occurred in two interviews). The mean length of interviews was 6 mins and 40 s. Twenty mothers and three fathers were interviewed. Twenty two parents were Irish and one parent was of Polish origin. Over half of participants were private patients (were in possession of private health insurance or paid for medical services). Data saturation was reached following 19 interviews. A further 3 interviews were carried out as per the Francis method [39]. Table 2 describes the characteristics of the participants and their children.

**Table 2:** Participant and child characteristics

	Age of parent (years)	Number of children	Age of children (years)
Mean	31.7	1.5	4.6
Range	26–40	1–4	1–15

A thematic coding inter-rater reliability score of 0.84 was obtained. Five themes emerged from the data: assessing and managing the fever; parental knowledge and beliefs regarding fever; knowledge source; pharmaceutical products; and initiatives.

### Assessing and managing the fever

Parents tended to assess the overall condition of the child before evaluating the illness: *“You wouldn’t go by temperature though, I’d judge her humour”* (Interview 2).

Parents differed with regard to pharmacological and non-pharmacological fever management: *“I'd probably leave it. No, I'd leave it a little bit and see - and you know, take it from there”* (Interview 13). Some would wait and see before medicating: *“I’d strip them down first, and maybe use a cold facecloth on their heads to see if that worked”* (Interview 4); while others used medication immediately. There was evidence that practices which have been designated as ineffective were still being used such as tepid sponging: *“I give them a spoon of Calpol or a cold cloth on the forehead”* (Interview 19).

### Parental knowledge and beliefs regarding fever

Temperatures that parents associated with fever ranged from 37.5 to 39 °C. Parents reported a range of temperatures to define normal temperature between 36 and 38 °C. Parents often believed that fever was a sign of something such as a cold, teething or an infection. Some parents believed that fever was beneficial: *“It’s basically - fever helps the body get rid of whatever is inside. Infections and stuff”* (Interview 3). The majority of parents believed fever was a normal childhood ailment: *“I think it’s just part of growing up and everyday things”* (Interview 13). A minority of parents perceived that they had limited knowledge regarding fever: *“Probably I think even for myself I would probably be misinformed on a lot of conditions and fever and what is actually normal and what is not normal, you kind of, I would just kind of guess maybe”* (Interview 11).

Parents had a variety of worries and concerns when their child had a fever. These worries and concerns were as a result of parental beliefs, attitudes and knowledge level regarding fever and febrile illness. These mainly centred on complications such as meningitis and convulsions: *“Yeah. I mean, that’s where sometimes that’d be the worry. Like, if it gets to such a level where there would be convulsions”* (Interview 21). Other concerns included the height of the fever, how quickly the fever rises, poor appearance of the child (including behavioural changes) and cause of fever. Some parents were not concerned by a low-grade temperature while others were immediately worried at any increase in temperature above their definition of normal temperature. Overall, parents worried if a high temperature persisted: *“But if it persisted I would tend to be nervous”* (Interview 17), particularly if medication did not decrease the temperature.

### Knowledge source

Parents’ main method of seeking help and sharing responsibility was to consult family members or a GP: *“First would be family, I would say, because I would be the youngest in my family so I have some olders that have kids”* (Interview 14). For a number of parents, information from family was given extra importance. Help-seeking incorporated parents seeking care for their child and also seeking knowledge from the GP regarding the symptoms. Some parents referred to telephoning the GP prior to presenting at the clinic to check whether it was necessary to attend. A minority of parents referred to using nurse-led phone lines or booklets provided by private health insurance companies. Parents also presented at out-of-hours services. No parent reported presenting at an ED without being referred there by their GP. The main reason selected for consulting GPs was to share responsibility with a professional: *“Normally what we use, we go to the doctor and they tend to give us advice and that’s kind of it then. I wouldn’t feel the need then to maybe second guess the doctor. If I think that it’s working, what they’ve suggested, I just tend to leave it at that”* (Interview 20).

There were mixed feelings on using the internet for information. Some parents felt that they could not always trust the information contained on internet sites. Others felt that if the information on a few websites concurred, then it was safe to use: *“I just kind of - I’d look at three or four of them (webpages), to be honest, and compare their answers”* (Interview 1). Some parents preferred webpages created for parents which used jargon-free terms.

Parents also relied on past experience to guide their management, together with getting to know the child: *“Finding out as time goes on, and getting to know the child, and know that this is not normal today, because she’s never behaved like this”* (Interview 8).

### Pharmaceutical products

The main pharmaceutical products used were Calpol® (paracetamol) and Nurofen® (ibuprofen) as parents believed these medications decrease high temperatures. The key reason for selecting a particular antipyretic was related to child attitude and preference with regard to the medication: *“Yeah, that's just because of her. She won't take anything else (Calpol®)”* (Interview 18). Certain parents believed that Nurofen® was more effective than Calpol® to reduce a high temperature. There was evidence that some parents alternated antipyretics as they believed this practice decreased high temperatures more efficiently than use of a sole agent. Parents indicated that they used both liquid and suppository form of medicines, however liquid forms were more commonly used.

There were contrasting attitudes with regard to forms of medication used, with several parents indicating that they did not like to use suppositories: *“No, I hate it (suppositories), and I'm not going to do that to my children”* (Interview 19). Others believed that suppositories were difficult to use. However, parental attitudes towards suppository use sometimes changed when the child had a particularly high temperature: *“I would use the suppository ahead of the liquid if it was over 38.5”* (Interview 9), *“…as a suppository form it gets done quickest, I think”* (Interview 10) or when recommended by doctors. Despite the awareness that suppositories were equally or more effective than liquid forms of antipyretics, parents’ attitudes towards suppositories resulted in reluctance to use them.

### Initiatives

Parents’ level of knowledge, beliefs and attitudes regarding fever resulted in an overall desire for initiatives: *“Oh yeah. Definitely. Where they're getting it at the moment only is the doctor’s rooms. Not being funny like which as I say, where do they end up? If you had a site to go to it’d be handy”* (Interview 14). The majority of parents were of the opinion that accessible, consistent information was necessary: *“I suppose there would be a need for it, for people who wouldn’t have any experience, especially people maybe with their first child or something like that”* (Interview 20). A variety of means of transmission for the information were suggested including booklets, apps, a leaflet, books or via the Public Health Nurse. Other suggestions included the provision of visual information: *“It’s something visual, if you could see the visuals, do you know”* (Interview 14). Parents suggested that they were often distracted when receiving information and therefore were of the opinion that information should be available in a form which could be accessible when their child was sick: *“I don’t know where else that you would access it because I don’t think that mothers, being first time or otherwise, have a lot of time to be reading stuff. A friend of mine said that her grandmother always said that first-time mums, you could be having a conversation with them, but they’re thinking about something else entirely. They’re not hearing you.”* (Interview 8).

## Discussion

The knowledge, attitudes and beliefs of parents of children with at least one child aged five years of age or under were identified through semi-structured interviews. Five themes emerged from the data: assessing and managing the situation; parental knowledge and beliefs regarding fever; knowledge source; pharmaceutical products; initiatives.

The data indicate that parents had a good level of knowledge regarding fever and febrile illness. However, a desire to seek information and share responsibility encouraged parents to seek the opinions of others, especially healthcare professionals with regard to the severity of symptoms and management strategies. Parents expressed a desire for simple, accessible information to be provided which could be referred to when required.

Most parents accurately described normal and febrile temperatures in line with previous research [45]. However, definitions of normal and febrile temperatures overlapped. This illustrates a general rather than a specific knowledge of temperature ranges. Parents’ ability to offer broad rather than specific information may be as a result of healthcare professionals assuming a higher baseline knowledge and therefore not providing precise temperature ranges with a description of whether the temperature is in the normal or febrile range. It could also be a result of parents receiving mixed messages from a variety sources including healthcare professionals, family, friends and internet-based sites. Healthcare professionals should be encouraged to express clear temperature ranges when describing normal and febrile temperatures to help communicate this information to parents. Healthcare professionals should also ensure that all messages given to parents regarding fever are based on standardised, evidence-based information to eliminate conflicting information.

Parents indicated that temperature alone was sometimes used as a trigger to administer antipyretics. Some parents in this study believed that fever should not automatically be treated in children who are not distressed, as supported by existing literature [9, 34, 46, 47]. Similar to previous research, parents believed that fever could cause adverse effects such as seizures [8]. However, attitudes of parents in this cohort showed no awareness that lowering the temperature or the use of antipyretic agents does not prevent febrile convulsions, which contrasts with existing literature [1, 9, 46–48]. Most febrile seizures occur at the onset of fever, hence explaining why prophylaxis is ineffective in most situations [48]. No parent in this cohort mentioned the side effects of paracetamol [46] documented in previous studies. Similar research conducted in Denmark indicated that parents were reluctant to use medication, possibly due to negative effects of medication [34], which was not illustrated in this cohort. This indicates that risk factors associated with antipyretics may not be a key priority for parents in Ireland when deciding to use antipyretics. A lack of parental information or misinformation regarding the inability of antipyretics to prevent febrile seizures, lowering temperature level not automatically preventing febrile seizures, and that antipyretics have side effects, may offer an explanation for the substantial use of antipyretics, as previous research has shown that antipyretics are some of the most commonly used drugs in children [49].

This study demonstrates that parental attitudes towards seeking information is possibly linked to a lack of confidence when managing fever. Parents with private health insurance referred to using resources which were made available by private health insurance companies such as nurse phone lines and books. Perhaps similar initiatives should made available to non-insured parents to decrease health disparities. Failure by parents to shoulder any diagnostic risk overloads health systems and subjects children to unnecessary worry and investigation [50], therefore initiatives to help parents deal with risks and uncertainty associated with fever and febrile illnesses need to be introduced to prevent or reduce over engagement with health services.

This study confirms that a high temperature is a trigger for parents to alternate their use of antipyretics. Health professionals frequently recommend treatment regimens for children with fever that either combine or alternate paracetamol and ibuprofen [51, 52]. However, guidelines recommend that paracetamol and ibuprofen should not be used together [1]. Healthcare professionals need to offer advice to parents which follows current guidelines to help reduce ambiguity surrounding the correct management of fever. There was no evidence that parents in this study believed that resolution of fever following administration of paracetamol or ibuprofen does not reflect the severity of the illness or that the severity of the illness does not correlate to the height of the temperature when a child is over 6 months old, which contrasts with existing literature [47].

Parents were reluctant to use suppositories, a finding which diverges from earlier research [53]. Parental negative attitude towards to the use of suppositories may be culturally linked as suppository use in other cultures is the preferred method of administration. Previous research indicated that 97 % of parents in an Icelandic study had used suppository forms of paracetamol for children and 56 % of participants exclusively used suppository forms of paracetamol [53].

Similar to previous research [5, 8, 34, 54], parents indicated that written information with the incorporation of visual aids would be a welcome addition to family-centred care. Parents illustrated that they wanted to have access to this information when their child is sick, to help guide and reassure them when managing the fever. This finding reflects parents perceived level of knowledge and/or confidence when managing fever in their children. Previous research has also shown that GPs are of the opinion that provision of enhanced information for parents in consulting rooms would decrease consultations due to fever [7]. Most healthcare professionals are conscious that fever in itself is not injurious to health, however, we need to do more to communicate the message to parents and carers [9]. Policy makers need to acknowledge the views of parents and GPs on this matter and provide information resources which parents can access to empower parents to manage their child’s illness.

As time allotted for health care visits decreases [55], the effectiveness of communication needs to be enhanced through increased initiatives, strategies, organised efforts and resources for parents. GPs already perceive that childhood fever consultations place a significant burden on their workload and that time-pressure can lead to frustration when examining children [7]. Therefore, opportunities to educate parents when they attend with their children for regular check-ups or vaccinations should be used to provide information on common childhood symptoms such as fever. Furthermore, Pharmacists are ideally located to provide timely, accurate and accessible support to parents which could alleviate the workload and time pressures for GPs. Every contact a parent makes with a healthcare professional should be used to impart information and provide knowledge to empower the parent. Policy makers should make materials available for effective health education which can be given to parents to take home. Collaboration between healthcare professionals and parents needs to grow in order to increase parental knowledge regarding definition, causes and management of fever and febrile illnesses [7, 12, 55, 56].

## Conclusion

This study demonstrates that while parents have general knowledge regarding fever, they lack an in-depth knowledge concerning the less obvious details of the symptom. In order to promote health, healthcare practitioners and policy makers need to acknowledge this information gap and target strategies to address the problem so that parents can become fully informed and empowered carers for their children.

### Limitations

As parents were concerned that they may lose their place in the queuing system if they spent too long at the interview this led to short interview duration. A greater number of mothers than fathers were interviewed which could make results of this research more applicable to the maternal rather than paternal viewpoint. However, no variations between the responses of mothers and fathers were observed. The public location and profession of the interviewer could have encouraged socially acceptable answers. In some clinics, a room was provided for interview. These interviews were usually longer and more in-depth information was obtained. However, no difference in ideas or data captured was observed between the interviews in public and private areas. Limited demographic information was collected about participants, therefore it was not possibly to discern group profiles (e.g. based on education level, health literacy or internet access).
